# Does Whistleblowing on Tax Evaders Reduce Ingroup Cooperation?

**DOI:** 10.3389/fpsyg.2021.732248

**Published:** 2021-10-06

**Authors:** Philipp Chapkovski, Luca Corazzini, Valeria Maggian

**Affiliations:** ^1^National Research University Higher School of Economics, Russian Federation, Moscow, Russia; ^2^Department of Economics and VERA, University of Venice “Ca’ Foscari”, Venice, Italy

**Keywords:** tax evasion, whistleblowing, ingroup cooperation, spillover effects, laboratory experiment JEL classification: H26, C90, D02 PsychoINFO classification: 2900, 4200

## Abstract

Whistleblowing is a powerful and rather inexpensive instrument to deter tax evasion. Despite the deterrent effects on tax evasion, whistleblowing can reduce trust and undermine agents’ attitude to cooperate with group members. Yet, no study has investigated the potential spillover effects of whistleblowing on ingroup cooperation. This paper reports results of a laboratory experiment in which subjects participate in two consecutive phases in unchanging groups: a tax evasion game, followed by a generalized gift exchange game. Two dimensions are manipulated in our experiment: the inclusion of a whistleblowing stage in which, after observing others’ declared incomes, subjects can signal other group members to the tax authority, and the provision of information about the content of the second phase before the tax evasion game is played. Our results show that whistleblowing is effective in both curbing tax evasion and improving the precision of tax auditing. Moreover, we detect no statistically significant spillover effects of whistleblowing on ingroup cooperation in the subsequent generalized gift exchange game, with this result being unaffected by the provision of information about the experimental task in the second phase. Finally, the provision of information does not significantly alter subjects’ (tax and whistleblowing) choices in the tax evasion game: thus, knowledge about perspective ingroup cooperation did not alter attitude toward whistleblowing.

## Introduction

Tax evasion and tax fraud represent a major concern all over the world^1^, subtracting fiscal resources that are needed to finance public goods and questioning the effectiveness and fairness of tax systems.

Whistleblowing by citizens has recently gained increased attention as an effective and viable strategy to contrast tax evasion. For instance, according to the IRS Whistleblower Office, between 2007 and 2016, information submitted by whistleblowers has helped the United States government to recover $3.4 billion of tax revenue^2^.

Despite the potential fiscal benefits of whistleblowing, the number of studies analyzing its determinants and socio-economic consequences is still limited. In this respect, while there is evidence showing that trust in the government represents an important determinant of the decision to blow the whistle on tax evaders (Antinyan et al., 2020) a research question that remains unexplored is whether whistleblowing can undermine the quality of social interactions within communities. As numerous studies have been shown, those who dare to report the norm violation or crime committed by their own group members are indeed under risk of being stigmatized by their communities (Woldoff and Weiss, 2010). Ostracism of snitchers goes far beyond socially vulnerable groups (such as ethnic minorities, prisons, or districts with high crime rate), including school classes (Morris, 2010) and police departments. Apart from the potential retaliation of the norm violator, whistleblowers also risk to be victim of actions of other members of their reference group, who usually prefer not to work with them (Reuben and Stephenson, 2013). In particular, even when anonymity is fully assured, the whistleblower’s actions might be perceived as undermining ingroup trust (Wallmeier, 2019), so that whistleblowing could negatively affect future group cooperation.

In this paper, we report results of a laboratory experiment aimed at: (i) investigating the effects of whistleblowing on tax evasion; and (ii) assessing its potential consequences on ingroup trust and cooperation.

Our experiment includes two consecutive phases. In the first phase, we implement a simple tax evasion game in which participants, randomly assigned to group of five members according to a fixed matching protocol, have to decide the amount of their income they want to report to the central authority in order to pay taxes. In case of auditing, if the declared income is lower than the actual one, the individual has to pay the back taxes on the undeclared income plus a fine.

In the second phase, participants play a generalized gift exchange game. In particular, subjects simultaneously decide how much of their endowment to send to other group members, knowing that the amount sent will be doubled by the experimenter.

We manipulate two main dimensions of our experimental design: the presence of a whistleblowing mechanism and the provision of information at the beginning of the first phase about the content of the experimental task in the second phase. Concerning the first dimension, we distinguish between *Whistleblowing* and *NoWhistleblowing* treatments. In the *Whistleblowing* treatments, after all income declaration choices have been made, each subject is given the possibility to blow the whistle on others so to increase their probability of being audited by the tax authority. Moving to the second manipulated dimension, in the *Information* treatment, information about the content of the experimental task in the second part is provided at the beginning of the experiment, while in the *NoInformation* treatment subjects learn about the second phase only at the end of the tax evasion game. Thus, the information manipulation allows us to investigate whether being aware about the forthcoming cooperative task in the second phase strategically affects the efficacy of whistleblowing and tax evasion in the first phase, making group subjects more reluctant to blow the whistle on other group members.

Our results are summarized as follows. First, whistleblowing is effective in reducing tax evasion as well as in improving the precision of tax auditing. Indeed, participants blow the whistle on ingroup members who misreport their income and the risk of being signaled to the tax authority increases the overall level of tax compliance. Second, we detect no statistically significant spillover effects of whistleblowing on ingroup cooperation in the subsequent generalized gift exchange game, with this result being unaffected by subjects’ information about the experimental task in the second part.

The rest of the paper is organized as follows. Section “LITERATURE REVIEW” summarizes the related literature while in Section “EXPERIMENTAL DESIGN” we introduce our experimental design and the experimental procedures implemented. In Section “RESULTS” we present our results and discuss possible explanations. Section “DISCUSSION” concludes and suggests directions for future research.

## Literature Review

In this study we investigate the existence and sign of cross-contexts spillover effects of whistleblowing on ingroup trust. Near and Miceli, 1985 (page 4) define whistle-blowing as “the disclosure by organizational members (former or current) of illegal, immoral, or illegitimate practices under the control of their employers, to persons or organizations that may be able to effect action”. This widely used definition refers to the hierarchical type of relations where the reported hold structurally more powerful positions than those who report (Loyens, 2013). The main focus of this paper is instead peer reporting whistleblowing, defined as “a lateral control attempts that occur when an in-group member discloses a peer’s wrongdoing to higher authorities outside the group” (Trevino and Victor, 1992). In the rest of the paper we will use the terms ‘whistleblowing’ and ‘peer reporting’ interchangeably.

Our paper relates to the recent and flourishing literature that investigates the within- or across-context spillovers of policy interventions, which focuses mostly on how they might affect prosocial norms and social preferences beyond those behaviors directly targeted by the institutions (Peysakhovich and Rand, 2016; dAdda et al., 2017; Galbiati et al., 2018; Ghesla et al., 2019). In the laboratory experiment by Engl et al. (2020), participants sequentially play two identical public good games, such that cooperation is institutionally enforced only in the first one. They find evidence of significant positive spillover effects of the institution, meaning that it increases cooperation also in the unregulated game, affecting preferences and beliefs about others’ attitude to cooperate. Furthermore, Galeotti et al. (2021) show how policy interventions can exert unintended behavioral effects that go beyond their original scope. More specifically, in their quasi-experiment, both fraudsters and non-fraudsters in public transport when exposed to ticket inspections were more likely to misappropriate money in a different unrelated context, providing evidence of negative spillover effects of deterrence institutions on intrinsic honesty.

Whether, and under which conditions, whistleblowing represents an effective instrument to curb tax evasion is an intriguing research question that is gaining increasing attention in recent years. Breuer (2013) experimentally investigates whether incentivization of whistleblowing is effective for fostering tax compliance and shows that whistleblowing is successful in limiting tax evasion, even without monetary incentives. Bazart et al. (2020) experimentally study the impact of a whistleblowing-based audit scheme upon taxpayers’ reporting decisions. They design an experiment aiming at comparing the relative efficiency of whistleblowing opportunities compared to a standard random-based audit scheme, keeping operating costs constant for the tax administration (neither rewards nor denunciation costs are considered). Their findings confirm that whistleblowing-based audit scheme decreases the monetary amount of evasion, improves the targeting of evaders and raises the tax levy. In their experimental study, Masclet et al. (2019) investigate the effect of whistleblowing programs on tax evasion providing information to participants on the use of the tax revenues in three dynamic treatments: (i) a baseline treatment where tax evaders are obliged to pay taxes on the undeclared income and a penalty if audited, (ii) an information treatment in which participants are also informed about the income declaration rates of all other group members and (iii) a denunciation treatment in which each participant has the possibility to blow the whistle on others. They find that monitoring alone does not increase the declared income while allowing for blowing the whistle decreases tax evasion; moreover, informing participants that the tax revenue was used to finance an environmental public good has no significant impact on either tax compliance or peer reporting. However, the role of information about other tax payers seems to affect the tax compliance rate according to a non-trivial relationship (see the corresponding section of the metastudy examining main factors affecting tax evasion Alm, 2019). On the one hand, if an individual knows that his neighbors are cheating with taxes, he will be more likely to evade taxes as well (Alm et al., 2017). On the other hand, the threat of public disclosure of tax evaders’ identity may serve as an effective deterrrent: the cross-cultural study run by Alm et al. (2017) reveals indeed that when the photos of tax evaders were shown to the rest of the group, full compliance raised from 38% to 57%.

Nyreröd and Spagnolo (2021) investigate the effects of introducing economic incentives to stimulate whistleblowing and show that rewarding whistleblowers is associated with a reduction in misbehaviors. Amir et al. (2018) extends the analysis to the indirect effects of the introduction of a whistleblowing program in 2013 in Israel to combat tax evasion. Their findings support the hypothesis that, despite the limited direct effect on tax collection, whistleblowing indirectly increases tax revenues through deterrence.

The effect of whistleblowing programs is not limited only to the tax evasion schemes. They are also proved to have a strong deterrent effect as an antitrust measure (Apesteguia et al., 2007; Hinloopen and Soetevent, 2008). The way a whistleblowing scheme is designed to fight against cartels is usually different from what is observed in tax compliance because, in contrast to the individual crime of tax evasion, the creation of a cartel implies a collusion between group members. Thus, a law maker has to show leniency toward whistleblowers, whose degree affects the effectiveness of the program (Chen and Rey, 2013), something which also depends on the intrinsic motives of the whistleblower (Heyes and Kapur, 2009). Buckenmaier et al. (2020) show that introducing the possibility to blow the whistle on others both reduces the probability that subjects collude and accept bribes and increases tax compliance. More importantly, they also document strong spillover effects of leniency programs, with a strong time persistence of the effects of the whistleblowing program after its removal. Our experimental study is aimed at shedding light on another potential spillover effect of whistleblowing. Indeed, as long as whistleblowing is interpreted as a non-cooperative institution that is mainly intended to punish other group members, institutionalizing the possibility of individuals to denounce each other’s wrongdoing might finally result in an erosion of ingroup trust, making coordination and cooperation for mutual benefit more difficult to achieve. Ingroup trust is indeed a necessary component of group cohesion (Fonseca et al., 2019), which in turn affects a group’s ability to successfully participate in cooperation and coordination games (Gächter et al., 2017). When an individual makes a decision about peer reporting, he might undermine this loyalty, lowering other members’ willingness to cooperate. However, the relations between group loyalty and norm violation are complex. On the one hand, loyalty can decrease norm violations within groups (Hildreth et al., 2016) while, on the other hand, people tend to perceive loyal but dishonest actions as more ethical than disloyal but honest ones (Hildreth and Anderson, 2018).

Whistleblowing has been also investigated in different contexts, including corruption and the work environment. In particular, depending on the level of interdependency of work tasks, the work environment represents a further important context in which ingroup trust and whistleblowing institutions are strongly related to each other (Lau and Liden, 2008). Concerning how whistleblowing affects, and is affected by, awareness about future interactions in the workplace, there are important papers that are close to ours. In a hierarchical framework, Wallmeier (2019) investigates the emergence of fraudulent whistleblowing. More specifically, in his laboratory experiment, a manager and an employee play a modified version of a trust game. Before interacting with the employee, the manager can engage in embezzlement, which in turn exerts a negative externality on a third party. The employee observes possible misbehavior and may report it to an external authority. He finds that both introducing an incentivized and an anonymous reporting mechanism increases fraudulent whistleblowing and discourages subsequent group cooperation. Finally, Reuben and Stephenson (2013) investigate a situation in which individuals have the opportunity to blow the whistle on those who lie for personal advantage and found that whistleblowers are indeed ostracized. However, differently from these papers, anonymity of the whistleblower is fully assured in our study, which in turn removes the possibility of ostracism and direct retaliation. In this respect, beside its deterrence effects, our experimental design is aimed at assessing the indirect effects exerted by whistleblowing in the tax evasion game of the first phase on the level of ingroup trust and cooperation in the different, generalized gift exchange context subjects participate in the second phase.

## Experimental Design

The experiment consists of two consecutive phases. In the first phase of the experiment, individuals participate in 10 rounds of a tax evasion game, while in the second phase they play a generalized gift exchange game for five rounds. In both phases, subjects always interact with the same group members. Indeed, at the beginning of the experiment, groups of five subjects are randomly formed and their composition is kept constant throughout the two phases.

In each round of the first phase of the experiment, each individual is assigned with a gross income expressed in ECUs (Experimental Currency Units). In particular, the gross income of each subject is an integer number that is randomly drawn from a uniform distribution between 100 and 240. Given her gross income, each subject chooses how much to declare to the central tax authority for tax payments, knowing that, on the declared amount, she will pay a flat tax rate of 30%. In each period, the declared income of one of the five group members is randomly selected (thus corresponding to a probability of 20%) and audited by the tax authority to verify its conformity with the gross income. If the subject under-declares her gross income, then, in addition to the due taxes on the gross income, she will pay a fine that is set equal to the evaded taxes (namely, the 30% of the difference between the gross and the declared income). If the subject fully declares her gross income, then the audit mechanism does not produce any further effect on her payoffs. Once the declaration choice is submitted, information about others’ gross and declared incomes is provided. Finally, at the end of every period, each subject is informed about her payoffs and whether her choice has been selected for auditing.

With respect to the *NoWhistleblowing* treatment, in the *Whistleblowing* treatment the only difference is that once all declaration choices are submitted and information about others’ gross and declared incomes is provided, each subject can blow the whistle on other group members. In particular, each subject is given the possibility to signal one of the four remaining group members to the tax authority. Then, the computer randomly selects one whistleblower. If the whistleblower effectively blew the whistle on one group member, then her choice is implemented, and the declared income of the signaled subject is audited. On the other hand, if the whistleblower decided not to blow the whistle on anybody, then, as in the *NoWhistleblowing* treatment, one of the group members is randomly selected and her declared income audited. Finally, no information is given to the audited subject on whether audit was due to random selection or to whistleblowing by other group members.

While most real-life leniency programs provide whistle-blowers with some indulgence for their own violations, our experimental design does not entail any bonuses in monetary or non-monetary form for those denouncing other tax evaders. This non-incentivized whistleblowing design is standard in tax evasion experiments [see, for instance, Bazart et al. (2020)], representing a conservative test to measure individuals’ propensity for blowing the whistle: if we observe peer reporting without extra motives, we expect such a behavior to occur even with a higher frequency when individuals are positively incentivized to do so. In a similar vein, in our experiment the tax revenues plus the fines are not returned back to the common pool. Masclet et al. (2019) experimentally compared peer-reporting (whistleblowing) treatments with and without positive externalities and found no difference in whistleblowing frequency when participants were informed that collected taxes were used to purchase carbon credits.

In the second phase of the experiment, participants play the generalized gift exchange game. In each of the five periods of the second phase, each subject receives an endowment of 100 ECUs and chooses how much to send to the remaining group members. Whatever she sends is doubled by the experimenter and distributed equally among the remaining four group members. Therefore, social welfare is maximized if everyone sends the maximum amount to peers. This game is a variation of the standard public good game where an individual share of investment to a public good is not returned to the initial investor. Unlike a strain of the experimental literature that uses the sequential gift exchange game (Charness, 1996; Charness and Haruvy, 2002), in our experiment participants have to make their choices simultaneously. Additionally, instead of providing a gift to one single member of their group (Kanitsar, 2019), in our design each individual provides a gift to all other group members. Besides allowing for very simple and short instructions, our choice to implement a generalized gift exchange game characterized by simultaneous decisions was driven by our research objective, namely to investigate whether having experienced a tax evasion game with or without the possibility to blow the whistle on other group members affect the individual’s beliefs about the overall level of cooperation of other players, and the individual decision to give as a consequence.

Apart from the inclusion of a whistleblowing stage, our experimental design also manipulates the provision of information about the content of the second phase before the tax evasion game is played. While in the *NoInformation* treatments, participants are informed about the second phase of the experiment only after completing the tax evasion game, in the *Information* treatments all participants learn, since the beginning of the experimental session, the content and instructions of the generalized gift exchange game of the second phase. The purpose of the information manipulation is to investigate whether tax evasion and attitude to blow the whistle are affected by subjects’ awareness about the fact that, in the subsequent phase, they will participate with their group members in game in which results strongly depend on the level of ingroup trust. Even if anonymity is fully assured, whistleblowing might indeed undermine ingroup trust, making cooperation in the generalized gift exchange game more difficult to achieve. By anticipating these considerations, individuals might therefore be more reluctant to blow the whistle on others, nullifying the effectiveness of whistleblowing in curbing tax evasion. The combination of the two manipulated dimensions generates results in a 2 × 2 design, and henceforth we will refer to the four treatments with the following labels: *NoWhistle_NoInfo*, *Whistle_NoInfo*, *NoWhistle_Info* and *Whistle_Info*.

### Experimental Procedures

The experiment was run between September and December 2019 at the CERME (Center for Experimental Research in Management and Economics) laboratory, in Ca’ Foscari University of Venice (Italy). 240 subjects (59% female), recruited through ORSEE (Greiner, 2015), participated in the experiment. Totally, we run 12 experimental sessions, with 60 subjects per treatment. Most of participants were undergraduate students (75.4%), enrolled in Economics (72.5%). Sessions were randomly assigned to treatments so that all participants within the same session were assigned to the same treatment and none participated in more than one treatment^3^.

The experiment was computerized by using o-Tree (Chen et al., 2016). Each session lasted around 75 min (including time for reading the instructions aloud, answering private questions, and paying) and the average payment was 13.5 euro, including a show-up fee of 3 euro. Although subjects participated in 15 rounds, to avoid wealth effects, only one of the 15 rounds was effectively used to determine final payments. Specifically, at the end of the experiment, the experimenter first selected one of two phases by tossing a coin. Then, given the phase, the experimenter randomly picked one of the corresponding rounds.

## Results

In this section, we present our results. Given the partner-matching protocol of our experiment, we perform both: (i) two-sample Mann–Whitney tests (MW) and (ii) Somers’ D median difference tests (Newson, 2002) at the group level, and we report results of (i) only unless the two tests give different results^4^.

### Tax Evasion Game

First, we describe the effect of whistleblowing on tax evasion.

**FIGURE 1:**
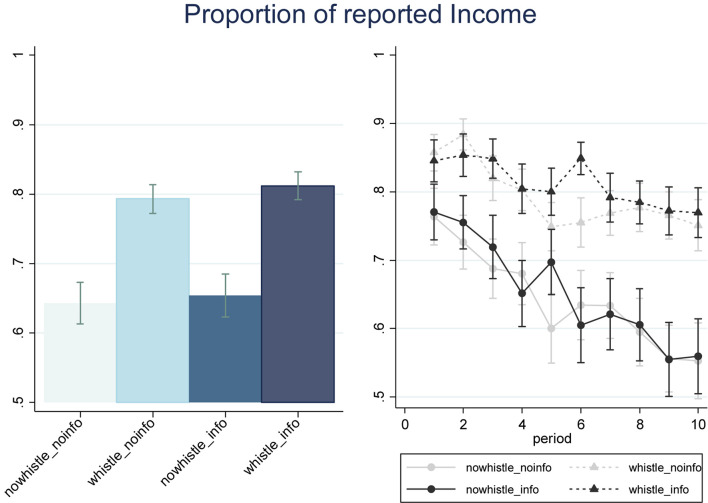
Proportions of gross incomes declared by subjects in the tax evasion game, by treatment (left-handed Panel) e by treatment and period (right-handed Panel), *N* = 240. Error bars, mean ± SEM.

In Figure 1, we show the proportions of gross incomes declared by subjects in the four treatments, both over the 10 periods of the first phase (left-handed Panel) and by period (right-handed Panel). Our data confirm that blowing the whistle is indeed effective in increasing the average proportion of reported income, being equal to 0.65 in the treatments in which subjects cannot signal others’ choices to the tax authority (*NoWhistle_NoInfo* and *NoWhistle_Info*) and equal to 0.80 in the treatments including the whistleblowing stage (*Whistle_NoInfo* and *Whistle_Info*), with this difference being highly significant (*p* = 0.001, MW). The same result is observed when making a pairwise comparison between *Whistle_Info* and *NoWhistle_Info* (*p* = 0.038, MW; *p* = 0.158, Somers’ D), as well as between *Whistle_NoInfo* and *NoWhistle_NoInfo* (*p* = 0.021, MW). Additionally, the decrease in the proportion of the reported income across periods is starker in absence of the deterrence mechanism than in treatments including the whistleblowing stage.

Finally, we see no effect of the information manipulation on the effectiveness of whistleblowing (*Whistle_Info* vs. *Whistle_NoInfo*, p = 0.862, MW).

**FIGURE 2:**
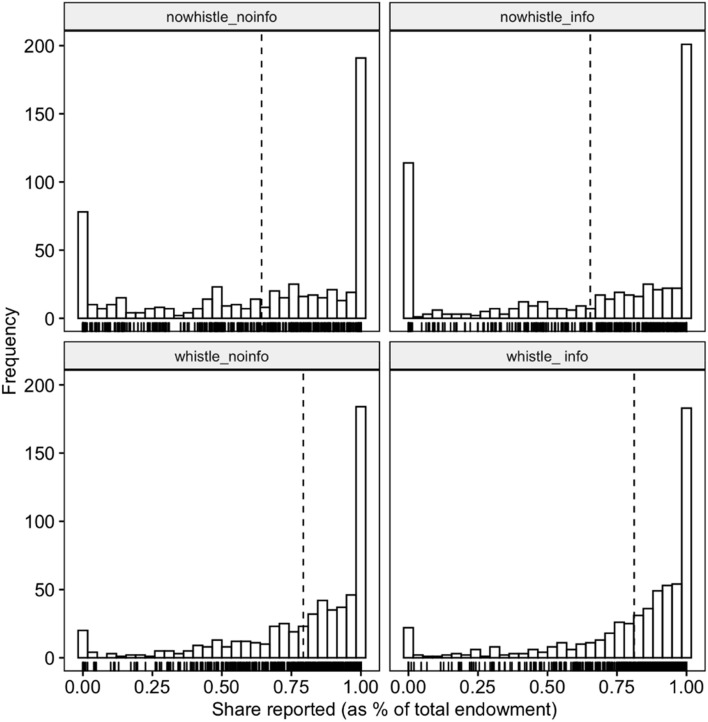
Frequency of proportion of reported income per treatment.

Figure 2 provides a more detailed picture of the frequencies of the relative reported share of income in each treatment. We observe that individuals are more likely to report an income equals to zero when whistleblowing is not allowed than in the *Whistle_NoInfo* and *Whistle_Info* treatments.

As it can be seen in Table 1, where we report the proportion of full compliers, intermediary compliers and full non-compliers, the most striking difference across treatments is indeed the substantial fall of full non-compliers as soon as the possibility to blow the whistle on others is introduced (from 11% and 18% respectively in the *NoWhistle_NoInfo and NoWhistle_Info* treatments to 2.8% and 3.5% in the *Whistle_NoInfo* and *Whistle_Info* treatments).

**TABLE 1:** Proportion of full compliers, intermediary and full non-compliers per treatment.

Treatment	Full compliers	Intermediary compliers	Full non-compliers
nowhistle_noinfo	29.3%	59.3%	11.3%
nowhistle_info	32.7%	49.3%	18.0%
whistle_noinfo	27.8%	69.3%	2.8%
whistle_ info	27.0%	69.5%	3.5%

In Table 2, we report parametric results from a series of Multilevel models, with standard errors that are clustered at both the group and subject level, using the proportion of gross incomes declared by subjects in each of the 10 rounds of the first phase as dependent variable^5^.

**TABLE 2:** The determinants of the proportion of income reported in the tax evasion game: Multilevel models, with standard errors clustered at both at the group and at the subject level.

Independent variables	Model 1	Model 2	Model 3	Model 4	Whistle	NoWhistle
Info	0.015	0.010	0.011	0.025	0.021	0.042
	(0.040)	(0.057)	(0.052)	(0.050)	(0.049)	(0.048)
Whistleblowing	0.155***	0.150***	0.138***	0.137***		
	(0.040)	(0.057)	(0.052)	(0.050)		
Endowment	−0.0003062**	–0.0003063	–0.0003618	–0.0003641	–0.0001289	–0.0006132
	(0.0001207)	(0.0001207)	(0.0001287)	(0.0001286)	(0.0001391)	(0.0002104)
	(0.000)	(0.000)	(0.000)	(0.000)	(0.000)	(0.000)
Period	−0.017***	−0.017***	−0.015***	−0.015***	−0.009***	−0.020***
	(0.002)	(0.002)	(0.002)	(0.002)	(0.002)	(0.003)
InfoXWhistleblowing		0.010	0.012	0.001		
		(0.080)	(0.073)	(0.070)		
Female			0.111***	0.061**	0.003	0.072
			(0.026)	(0.027)	(0.024)	(0.044)
Proportion_report_prev_period			0.105***	0.104***	0.190***	0.099***
			(0.022)	(0.022)	(0.032)	(0.031)
Prev_audited			−0.054***	−0.053***	0.047***	−0.149***
			(0.013)	(0.013)	(0.015)	(0.022)
Economics				−0.058**	–0.010	−0.091*
				(0.028)	(0.022)	(0.047)
Income_family				0.007	0.001	0.016
				(0.011)	(0.009)	(0.020)
Relative_wealth				0.003	0.009	–0.002
				(0.012)	(0.010)	(0.019)
Perceived_tax				−0.012*	0.011**	−0.038***
				(0.007)	(0.006)	(0.012)
Fair_tax				0.020**	0.0000744	0.046***
				(0.009)	(0.0082447)	(0.015)
Risk_audit				0.001	0.005	–0.002
				(0.006)	(0.005)	(0.010)
Risk_level				−0.019***	−0.019***	−0.027***
				(0.005)	(0.004)	(0.009)
Reciprocal_evasion				–0.008	–0.001	−0.024**
				(0.007)	(0.006)	(0.011)
Tax_Morality				–0.004	–0.005	–0.007
				(0.005)	(0.004)	(0.009)
Ineff_gov				0.006	–0.005	0.006
				(0.006)	(0.005)	(0.011)
High_tax				–0.005	−0.011**	–0.000
				(0.006)	(0.005)	(0.011)
Trust					0.003	−0.026**
					(0.006)	(0.011)
Help_others					0.029***	0.011
					(0.007)	(0.011)
Constant	0.787***	0.790***	0.656***	0.835***	0.558***	1.197***
	(0.041)	(0.046)	(0.049)	(0.090)	(0.083)	(0.175)
Observations	2400	2400	2160	2160	1080	1080
Log likelihood	–141.753	–141.746	–131.309	–117.171	257.351	–218.791
Wald chi2	133.505	133.523	169.087	211.584	161.056	170.058
*p*	0.000	0.000	0.000	0.000	0.000	0.000

Table 2 reports estimates of a series of Multilevel regression models. The dependent variable is the reported proportion of income in each period of the tax evasion game. Clustered standard errors at the group level and at the individual level appear in parentheses. ***, ** and * indicate significance at the 1% level, 5% level and 10% level, respectively.

In Model 1, *Endowment* takes a value from 100 to 240 (in integer numbers). *Info* is equal to one in the treatments in which information about the second phase of the experiment was provided prior to the beginning of the first phase and 0 otherwise. Similarly, *Whistleblowing* takes a value of 1 in the treatments in which participants were allowed to blow the whistle on other ingroup members in the tax evasion game of the first part of the experiment, and 0 otherwise. *Period* is a time counter, and it is introduced in the regressions to account for the effect of experience in the tax evasion game. Model 2 is augmented by adding the interaction term *InfoXWhistleblowing*.

Model 3 includes participants’ gender and information about the previous period. In particular, *Proportion_report_prev_period* stands for the individual proportion of income reported in the previous period, while *Audited_prev_period* consists in a binary variable indicating whether, in the previous period, the participant was audited or not.

Finally, in Model 4, we add *Economics*, which takes a value of 1 if the participants’ field of study is Economics and 0 otherwise, as well as a series of categorical variables extracted from the post experimental questionnaire^6^. Previous studies (Jackson and Milliron, 1986; Richardson, 2006) have indeed shown how both “demographic (i.e., gender), “economic” (such as income level and marginal tax rates) and “behavioral” (such as fairness and tax morale) characteristics can motive tax evasion so we controlled these factors through a series of independent variables. More specifically, to take into consideration that members of high income families might be more likely to evade taxes as well as the effects of increasing marginal tax rates on income declarations, we include *Income_family*, *Relative_wealth* and *Perceived_tax* in our regression. Both *Income_family* and *Relative_wealth* take a value from 1 (very low) to 10 (very high) and define the participant’s perception of the income of her own family as well as her perception of the relative position of the family’s income with respect to the average Italian family, respectively, while *Perceived_tax* takes a value from 1 to 12 and expresses the perceived tax rate paid by the participant, in 5% income brackets (with 1 being “less than 10%” and 12 being “above 60%”). On the same vein, *High_tax* measure the strength of the subject’s belief on whether the tax rate affects individual willingness to pay taxes.

Given the negative relationship with fairness and tax evasion (Richardson, 2006), we also add *Fair_tax*, which indicates which tax rate would be considered as fair. Attitude toward risk might affect tax evasion when in presence of audit schemes and penalties, the variable *Risk_level* thus measures individual risk aversion and takes a value from 0 to 10, with higher numbers expressing lower levels of risk aversion. In order to control for the subject’s attitude toward tax evasion, we include *Risk_audit, Reciprocal_evasion* and *Ineff_gov* as covariates in the regression. The three variables indicate how strongly the subject agrees on a 10-point scale (with 1 indicating complete disagreement and 10 complete agreement) with the statement that citizens do not pay taxes if they perceive that the audit risk is low, other citizens do not pay taxes, and collected taxes are inefficiently implemented, respectively. Expecting tax morale to possibly negatively affect tax evasion (Torgler, 2003) we include as regressor *Tax_morality, which* measures the strength of the subject’s belief on whether morality affects individual willingness to pay taxes, while we also control for the level of perceived trust (*Trust*) and concern about helping others as a moral duty (*Help_others*).

From Model 1, whistleblowing significantly increases the proportion of reported income and, therefore, represents a valid instrument to limit tax evasion^7^. Differently, the effect of providing information about the second phase of the experiment before letting subjects to declare their income in the tax evasion game does not affect the amount of evaded taxes. Looking at Models 2 to 4, the interaction term between Whistleblowing and Info never reaches significance, meaning that the proportion of income reported by participants when they are allowed to blow the whistle is not affected by being aware about the gift exchange game in the second phase of the experiment. Although the coefficient of the endowment is significant at the 5% level in Model 1, it presents a small magnitude, suggesting that it exerts only limited effects on participants’ decision to evade taxes.

As participants gain experience in the tax evasion game, they are less likely to fully report their income, as shown by the significant and negative coefficient of the time trend in all models.

Model 3 further analyses the dynamic pattern followed by choices in the tax evasion game. The proportion of reported income is positively correlated across periods and being audited in the previous period decreases the amount evaded in the current one. As expected, the level of risk aversion is significant and negatively correlated with tax evasion: an increase of one unit in risk propensity decreases the proportion of reported income by about 0.02.

In order to better investigate the effects of being audited on the subsequent choices in the tax evasion game, the last two columns of Table 2 focus on the sessions with and without whistleblowing, separately. We find evidence of the bomb-crater effect of tax audits (Mittone et al., 2017) only in the *NoWhistleblowing* treatments while, as expected, in the Whistleblowing sessions being audited in the previous period significantly increases the proportion of income reported in the current period, as it suggests participants that other in-group members might have blown the whistle on them. Interestingly, as shown by the coefficient of *Help_others* in the model focusing on the sessions with Whistleblowing, the more individuals think that helping others represents a moral duty, the higher the proportion of income reported, underlying the importance of moral values in determining tax evasion.

### Generalized Gift Exchange Game

Our aim is to identify whether allowing individuals to blow the whistle on others in the tax evasion game and the information about the subsequent phase of the experiment exerted any effect on their contribution decisions in the generalized gift exchange game in the second phase. On average, participants contributed 24.75 tokens in the *Whistleblowing* treatments and 33.13 tokens in the *NoWhistleblowing* treatments. Thus, whistleblowing tends to reduce cooperation in the subsequent game, though this effect is not significant (*p* = 0.143, MW; *p* = 0.058, Somers’ *D*-test, 48 clusters).

**FIGURE 3:**
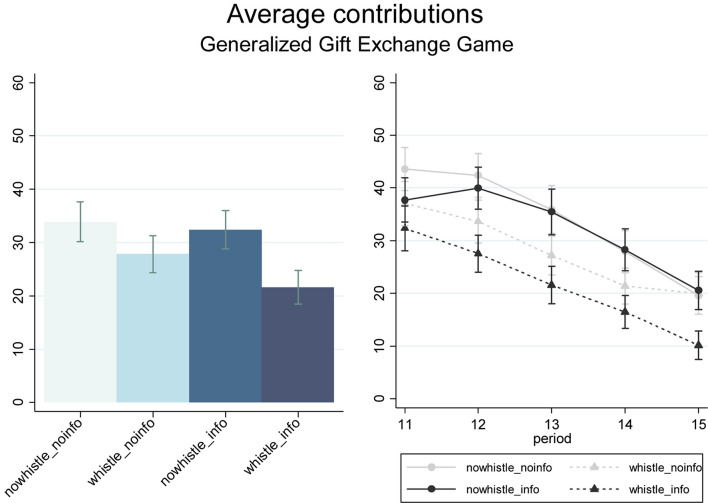
Average contributions in the Generalized Gift Exchange Game by treatment (left-handed Panel) e by treatment and period (right-handed Panel). Error bars, mean ± SEM.

In Figure 3, we report the average contribution in the *Whistleblowing* and *NoWhistleblowing* treatments, respectively. Allowing individuals to blow the whistle on others results in a slight reduction of contributions in the second phase of the experiment, in particular in the setting in which subjects receive information about the generalized gift exchange game before making their tax evasion choices (*p* = 0.133, MW; *p* = 0.078, Somers’ D). Instead, we document no significant effects in the setting in which the information about the task in the second phase is provided only at the end of the tax evasion game (*p* = 0.453, MW).

In Table 3, we report a series of multilevel models with standard errors that are clustered at both the group and subject level and where the dependent variable is the number of tokens contributed to the Generalized Gift Exchange Game^8^.

**TABLE 3:** Multilevel regressions. Amount contributed in the Generalized Gift Exchange game.

Independent variables	Model 1	Model 2	Model 3	Model 4	Whistle	NoWhistle
Whistleblowing	−8.408**	–6.030	1.026	1.241		
	(4.075)	(5.743)	(2.301)	(2.305)		
Info	–3.865	–1.487	1.302	1.816	−3.572*	1.532
	(4.075)	(5.743)	(2.143)	(2.155)	(2.167)	(2.173)
Period	−5.261***	−5.261***	−2.416***	−2.430***	−1.970*	−3.479***
	(0.439)	(0.439)	(0.712)	(0.710)	(1.029)	(0.982)
InfoXWhistleblowing		–4.757	–3.649	–4.272		
		(8.122)	(3.030)	(3.039)		
Contribution_prev_period			0.510***	0.504***	0.448***	0.523***
			(0.026)	(0.026)	(0.038)	(0.036)
Group_contribution_prev_period			0.260***	0.263***	0.157**	0.309***
			(0.044)	(0.044)	(0.072)	(0.056)
Proportion_report_1st_part			0.480	–0.882	–4.200	–0.634
			(3.628)	(3.694)	(7.766)	(4.344)
Group_proportion_report_1st_part			–7.800	–7.419	6.839	−14.775**
			(5.347)	(5.382)	(9.614)	(6.931)
Female			1.217	0.713	0.885	–0.647
			(1.606)	(1.625)	(2.277)	(2.328)
N_audited			0.093	0.064	–1.266	1.312
			(0.632)	(0.639)	(0.944)	(0.958)
Economics				–2.393	−6.189**	1.150
				(1.757)	(2.439)	(2.538)
Trust				0.080	0.524	0.026
				(0.436)	(0.604)	(0.642)
Help_others				0.700	0.593	0.663
				(0.461)	(0.669)	(0.637)
Tax_morality				–0.206	–0.079	–0.413
				(0.294)	(0.391)	(0.448)
Constant	103.457***	102.267***	39.025***	38.174***	32.499*	52.396***
	(6.710)	(7.004)	(11.123)	(11.748)	(17.167)	(16.167)
Observations	1200	1200	960	960	480	480
Log likelihood	–5581.4845	–5581.3136	–4390.5422	–4388.0923	–2184.389	–2192.5022
Wald Chi2	148.792	149.172	646.418	654.638	229.456	423.267
*p*	0.000	0.000	0.000	0.000	0.000	0.000

Table 3 presents the coefficients from a series of Tobit regressions left-censored at zero. The dependent variable is the amount contributed in each period of the generalized gift exchange game. Clustered standard errors at the session level appear in parentheses. ***, ** and * indicate significance at the 1% level, 5% level and 10% level, respectively.

In order to investigate whether allowing individuals to blow the whistle on others in the tax evasion game affects their contributions in the second phase, in Model 1 we include *Whistleblowing*, *Info* and *Period* as regressors. We observe that whistleblowing is indeed marginally significant in decreasing ingroup contributions in the gift exchange game. However, the effect disappears when information about the second phase of the experiment is not provided at the beginning of the experimental session, as shown by the coefficient of the variable *Whistleblowing* in Model 2.

In Model 3, we also add *Contribution_prev_period*, which stands for the individual contribution in the previous period, and *Group_contribution_prev_period*, that consists in a continuous variable expressing the average contributions of the remaining 4 group members in the previous period. We find a strong evidence in favor of in group reciprocity, whereby the average contribution made by a subject increases in the average number of tokens contributed by group members in the previous period. *Proportion_report_1st_part*, *Group_proportion_report_1st_part* and *N_audited* are built upon subjects’ behavior in the tax evasion game, and respectively indicate subject’s average reported income, the average income reported by the remaining 4 group members, and the number of times the participant was audited. Estimates indicate that results in the first phase of the experiment do not exert significant effects on the decisions in the gift exchange game. Similarly, Model 4 suggests that both the individual level of trust and willingness to help others do not significantly affect participants’ contributions.

Finally, in the last two columns of Table 3, we restrict our analysis on the *Whistleblowing* and *NoWhistleblowing* treatments. It is worth noticing that, when whistleblowing is introduced, providing information about the gift exchange game before playing the tax evasion game decreases contributions in the second phase, as shown by the negative and marginally significant coefficient of *Info*. Surprisingly, in the *NoWhistleblowing* sessions, the average income reported by the other 4 group members in the tax evasion game has a negative effect on individual contribution in the gift exchange game.

## Discussion

In this paper, we investigated the interaction between ingroup cooperation and whistleblowing. Stemming from the previous literature, we conjectured that whistleblowing may have exerted some unintended adverse effects, undermining the group morale, and compromising its ability for collective actions. If that would be the case, then even the positive effect the whistleblowing might have on tax payments could be outweighed by negative externalities of such institution.

Our results reject the existence of adverse spillover effects from the tax evasion game to the generalized gift exchange game: although the whistleblowing somewhat discouraged contributions in the generalized gift exchange game, when controlling for other factors this difference is not significantly different from zero.

Moreover, the main driving force behind our experiment was to observe whether the shadow of the future cooperation deter participants from blowing the whistle on tax evaders. Indeed, if whistleblowing is perceived as that, it would be the case that this can be one of the mechanisms that explain the reluctance of agents to blow the whistle. Being aware that whistleblowing would suppress the ingroup cooperation, the rational profit-maximisers would avoid to report tax evaders within their group. The results of our experiments do not confirm this intuition.

These results are good news for policy makers who try to promote whistleblowing as a means of horizontal control to fight the tax evasion or other norm-violating behavior. However, the lack of the effect may mean that we need to consider some other uncounted factors. For instance as Kennedy and Schweitzer (2018) have shown, whistleblowers are generally perceived as more trustworthy than individuals who stayed idle. Since these two effects push the cooperation rate to the opposite direction the net effect is hard to predict.

Additionally, as in most experimental studies, our study abstracts away from many elements of real life in order to cleanly identify the specific links between tax evasion, whistleblowing and cooperation. While in our experiment tax evaders are asked to pay a fine if they got caught, it would be interesting to allow participants to track the identities of ingroup members from round to round so to investigate the role of reputational considerations when evading taxes. Similarly, in our experiment, retaliation against whistleblowers is not possible, a phenomenon that might indeed refrain individuals from denouncing others’ wrongdoing. Finally, in our experiment the money collected through taxes are not meant to finance the provision of a public good. In such a situation, the benefits from higher levels of tax compliance due to whistleblowing might outweigh the possible decline in future cooperation. Future studies might evaluate the effects of these additional factors, in a framework where adopting a broader view in evaluating the efficacy of an institution allows to inform policies on the complex dynamics between tax evasion, whistle blowing and ingroup cooperation.

## Data Availability Statement

The raw data supporting the conclusions of this article will be made available by the authors, without undue reservation.

## Ethics Statement

Ethical review and approval was not required for the study on human participants in accordance with the local legislation and institutional requirements. The patients/participants provided their written informed consent to participate in this study.

## Author Contributions

All authors listed have made a substantial, direct and intellectual contribution to the work, and approved it for publication.

## Conflict of Interest

The authors declare that the research was conducted in the absence of any commercial or financial relationships that could be construed as a potential conflict of interest.

## Publisher’s Note

All claims expressed in this article are solely those of the authors and do not necessarily represent those of their affiliated organizations, or those of the publisher, the editors and the reviewers. Any product that may be evaluated in this article, or claim that may be made by its manufacturer, is not guaranteed or endorsed by the publisher.

## Footnotes

1 According to the most recent United States Internal Revenue Service tax gap report (Internal Revenue Service, 2019), the average annual gross tax gap was of $441 billion in tax years 2011–13 (slightly over 16 percent of total tax liability). In 2016, the VAT gap in Europe was estimated to be equal to EUR 147.1 billion, 12.3% of the total expected VAT revenue (Internal Revenue Service, 2019).

2 2016 Annual Report to the Congress of the Internal Revenue Service (https://www.irs.gov/pub/irs-prior/p5241–2017.pdf), retrieved on November 2, 2018.

3 In Supplementary Appendix Table A3 in the Supplementary Appendix we report the per-treatment main socio-demographic characteristics of our sample.

4 When performing the Mann–Whitney U-test, we average data at the group level and treat each group as an independent observation. The rank-order statistics Somers’ D looks at the individuals’ choices accounting for the presence of clusters at the group level (each experimental session included groups) in the data.

5 See Supplementary Appendix Table A.1 in the Supplementary Appendix for the results of a series of Tobit models (with left and right censoring at 0 and 1, respectively) with errors clustered at the group level. Results remain virtually unchanged across specifications.

6 The questionnaire (originally written in Italian) is reported in the Supplementary Appendix.

7 In Supplementary Appendix Table A4 in the Supplementary Appendix we provide a more detailed analysis of the whistleblowing behavior, defined as the per period number of whistleblower’s signals (from 0 to 4) on a group member as a function of her relative proportion of reported income within the group.

8 See Supplementary Appendix Table A.2 in Supplementary Appendix A for the results of a series of Tobit models, left censored at zero, with clustered standard errors at the group level. Results are almost unchanged. The only remarkable difference relies on the effect of N_audited. In the *Whistleblowing sessions*, the higher the number of times an individual was audited in the tax evasion game (and the higher the number of whistleblowers’ signals on the subject), the lower her contributions in the gift exchange game is. The opposite effect is instead observed in the *NoWhistleblowing* sessions, suggesting that being audited might have an educative effect on future cooperation.
