# Supplementary material for: Does Whistleblowing on Tax Evaders Reduce Ingroup Cooperation?
Source: Front Psychol. 2021 Oct 6;12:732248. doi: 10.3389/fpsyg.2021.732248 (PMC8526860; doi:10.3389/fpsyg.2021.732248)
Supplement: Supplementary file 1 [file Table_1.pdf]

Does Whistleblowing on Tax Evaders Reduce Ingroup Cooperation?: Supplementary information

Philipp Chapkovski[1] Luca Corazzini[2] Valeria Maggian[3]

Appendix A - Additional table and figures

Table A.1 Tobit models, clustering errors at the group level. Reported proportion of income in each period of the tax evasion game.

**Independent variables**	Model 1	Model 2	Model 3	Model 4	Whistle	NoWhistle
Info	0.013	0.007	0.001	0.013	0.018	0.030
	(0.063)	(0.094)	(0.053)	(0.051)	(0.035)	(0.062)
Whistleblowing	0.185***	0.180**	0.071	0.075*		
	(0.063)	(0.080)	(0.044)	(0.045)		
Endowment	-.0005054*	-.0005073*	-0. 0006781**	-0. 0007014**	-0.0004218	-0.0002104**
	(0.0002825)	(0.000281)	(0.0003184)	(0.0003148)	(0.000346)	(0.0005529)
Period	-0.026***	-0.026***	-0.010***	-0.011***	-0.007***	-0.016***
	(0.004)	(0.004)	(0.003)	(0.003)	(0.002)	(0.006)
InfoXWhistleblowing		0.011	0.010	0.004		
		(0.125)	(0.068)	(0.070)		
Female			0.095***	0.051*	0.021	0.059
			(0.028)	(0.029)	(0.025)	(0.062)
Proportion_report_prev_period			0.733***	0.683***	0.659***	0.708***
			(0.060)	(0.056)	(0.062)	(0.093)
Audited_prev_period			-0.061	-0.059	0.116***	-0.292***
			(0.038)	(0.038)	(0.022)	(0.061)
Economics				-0.050*	0.020	-0.147**
				(0.030)	(0.028)	(0.069)
Income_family				0.002	-0.002	0.019
				(0.009)	(0.011)	(0.019)
Relative_wealth				0.009	0.017	0.004
				(0.010)	(0.011)	(0.016)

[1] National Research University Higher School of Economics, Russian Federation . E-mail to: fchapkovskiy@hse.ru
[2] Department of Economics and VERA, University of Venice "Ca' Foscari", Cannaregio 873, Fondamenta San Giobbe, 30121 Venice, Italy. E-mail to: luca.corazzini@unive.it
[3] Department of Economics and VERA, University of Venice "Ca' Foscari", Cannaregio 873, Fondamenta San Giobbe, 30121 Venice, Italy. E-mail to : valeria.maggian@unive.it

Acknowledgments: Financial support from "Fondi Primo Insediamento", University of Venice "Ca' Foscari" is gratefully acknowledged. The contribution of Philipp Chapkovski was developed within the framework of the Basic Research Program at the National Research University Higher School of Economics (HSE).

Perceived_tax				-0.012	0.004	-0.036*
				(0.009)	(0.007)	(0.020)
Fair_tax				0.015	-0.002	0.038
				(0.011)	(0.008)	(0.024)
Risk_audit				-0.002	-0.001	-0.006
				(0.005)	(0.004)	(0.013)
Risk_level				-0.020***	-0.019***	-0.033***
				(0.006)	(0.005)	(0.011)
Reciprocal_ev asion				-0.010	-0.002	-0.030*
				(0.006)	(0.005)	(0.015)
Tax_Morality				0.002	0.00046	-0.005
				(0.005)	(0.0042239)	(0.012)
ineff_gov				0.006	0.001	0.011
				(0.005)	(0.005)	(0.012)
High_tax				-0.003	-0.012**	0.001
				(0.007)	(0.005)	(0.012)
Trust					-0.006	-0.044**
					(0.006)	(0.021)
Help_others					0.033***	0.020
					(0.011)	(0.017)
Constant	0.931***	0.934***	0.331***	0.541***	0.248**	1.079***
	(0.062)	(0.064)	(0.068)	(0.109)	(0.113)	(0.255)
Observations	2400	2400	2160	2160	1080	1080
Pseudo R-squared	0.035	0.035	0.222	0.236	0.436	0.210
F	14.337	12.268	36.047	22.259	40.962	13.386
P	0.000	0.000	0.000	0.000	0.000	0.000

Note: Table A.1 presents the coefficients from a series of Tobit models left-censored at zero and right-censored at one with errors clustered at the group level. The dependent variable is the reported proportion of income in each period of the tax evasion game. ***, ** and * indicate significance at the 1% level, 5% level and 10% level, respectively.

Table A2. Tobit models left-censored at zero, clustering errors at the group level. Amount contributed in the Generalized Gift Exchange game.

**Independent variables**	**Model 1**	**Model 2**	**Model 3**	**Model 4**	**Whistle**	**NoWhistle**
Whistleblowing	-10.104*	-6.654	2.350	2.413		
	(5.423)	(7.554)	(3.115)	(3.128)		
Info	-5.529	-2.083	1.694	2.194	-6.210*	1.398
	(5.462)	(9.153)	(3.513)	(3.430)	(3.339)	(3.008)
Period	-7.859***	-7.862***	-4.518***	-4.550***	-4.531***	-5.390***
	(0.897)	(0.898)	(0.967)	(0.978)	(1.391)	(1.213)
InfoXWhistleblowing		-6.977	-5.883	-6.796		
		(10.827)	(4.394)	(4.427)		
Contribution_prev_period			0.613***	0.604***	0.527***	0.630***
			(0.055)	(0.054)	(0.095)	(0.060)
Group_contribution_prev_period			0.369***	0.372***	0.213***	0.444***
			(0.063)	(0.062)	(0.074)	(0.073)
Proportion_report_1st_part			7.107	5.490	-2.744	8.155
			(7.605)	(7.420)	(14.789)	(8.808)
Group_Proportion_report_1st_part			-10.845*	-9.790	6.442	-14.352**
			(6.389)	(6.484)	(12.842)	(7.137)
Female			4.770**	4.160*	4.404	2.058
			(2.127)	(2.172)	(2.866)	(2.962)
N_audited			0.392	0.355	-2.264**	2.903***
			(0.827)	(0.860)	(0.985)	(1.069)
Economics				-1.665	-8.027**	4.970
				(2.611)	(4.053)	(3.764)
Trust				0.381	1.159	0.253
				(0.752)	(1.349)	(0.924)
Help_others				0.988	0.821	0.838
				(0.628)	(0.825)	(0.849)
Tax_morality				-0.557	-0.341	-0.906
				(0.383)	(0.601)	(0.587)
Constant	131.627***	129.951***	47.764***	45.772***	54.565***	50.354**
	(11.392)	(12.142)	(14.480)	(15.170)	(18.675)	(19.931)
Observations	1200	1200	960	960	480	480
Pseudo R-squared	0.012	0.012	0.060	0.061	0.047	0.075
F	29.432	22.746	43.664	34.499	16.917	46.674
p	0.000	0.000	0.000	0.000	0.000	0.000

Note: Table A.2 presents the coefficients from a series of Multilevel regression models, clustering both at the individual and group level. The dependent variable is the individual contribution in each period of the generalized gift exchange game. ***, ** and * indicate significance at the 1% level, 5% level and 10% level, respectively.

Table A3. Socio-demographic characteristics of the participants across treatments

	nowhistle_noinfo	nowhistle_info	whistle_noinfo	whistle_ info
**Age**				
Mean	21.78	21.38	21.20	21.28
St.deviation	2.96	2.17	2.63	2.76
**Gender**				
Female	56.67% (34)	58.33% (35)	60.00% (36)	61.67% (37)
Male	43.33% (26)	41.67% (25)	40.00% (24)	38.33% (23)
**Year of study**				
Primo (triennale)	28.33% (17)	15.00% (9)	18.33% (11)	25.00% (15)
Secondo (triennale)	21.67% (13)	25.00% (15)	25.00% (15)	21.67% (13)
Terzo (triennale)	28.33% (17)	33.33% (20)	31.67% (19)	28.33% (17)
Primo (specialistica)	10.00% (6)	13.33% (8)	13.33% (8)	8.33% (5)
Secondo (specialistica)	11.67% (7)	13.33% (8)	11.67% (7)	16.67% (10)
**Share of Economics students**				
	78.33% (47)	68.33% (41)	70.00% (42)	73.33% (44)
**Occupational status**				
No	40.00% (24)	36.67% (22)	30.00% (18)	50.00% (30)
Part-time	41.67% (25)	41.67% (25)	53.33% (32)	40.00% (24)
Full-time	18.33% (11)	21.67% (13)	16.67% (10)	10.00% (6)

In Table A4 we provide a more detailed analysis of the whistleblowing behavior. More specifically, Table A4 reports the per period number of whistleblower's signals (from 0 to 4) on a group member as a function of her relative proportion of reported income within the group. We first observe that while in the *Whistle_info* treatment in 19% of cases (114/600) participants decide not to blow the whistle at all, in the *Whisle_NoInfo* treatment the percentage decreases to 12% (72/600). Thus, being aware of future cooperative environments seem to make individuals more reluctant to blow the whistle on others, although the effect does not reach statistical significance (p=0.127, Somers' D, 24 clusters). In most of the cases, participants blow the whistle on the group member(s) who declared the lowest or the second lowest proportion of income within their group, namely 179 out of the 436 whistleblowing decisions made in the *Whistle_NoInfo* treatment (41%) and 163 out of 369 in the *Whistle_Info* treatment (44.2%). However, a non-negligible number of whistleblowing choices were addressed to those subjects fully reporting their income or declaring the highest proportion of their income within their group. Specifically, they were signalled to the central authority by at least 1 of their ingroup members 51 times (11.7%) in the *Whistle_NoInfo* and 41 times (11.1%) in the *Whistle_Info* treatments, respectively.

Table A4. Per period number of whistleblowers' signals on a group member as a function of her relative proportion of reported income.

	**Whistle_NoInfo treatment**						**Whistle_Info treatment**					
	**Number of whistleblowers' signals**						**Number of whistleblowers' signals**					
**Relative proportion of reported income within group**	0	1	2	3	4	**Total**	0	1	2	3	4	**Total**
Lowest proportion of reported income	7<br>2.31	19<br>11.59	27<br>45.00	39<br>81.25	23<br>92.00	115<br>19.17	11<br>3.24	10<br>7.46	33<br>58.93	33<br>82.50	27<br>90.00	114<br>19.00
Joint (2 individuals) lowest proportion of reported income*	1<br>0.33	3<br>1.83	3<br>5.00	1<br>2.08	0<br>0.00	8<br>1.33	2<br>0.59	2<br>1.49	2<br>3.57	1<br>2.50	1<br>3.33	8<br>1.33
Second lowest proportion of reported income	42<br>13.86	41<br>25.00	15<br>25.00	6<br>12.50	2<br>8.00	106<br>17.67	56<br>16.47	33<br>24.63	14<br>25.00	5<br>12.50	2<br>6.67	110<br>18.33
Joint (2 individuals) second lowest proportion of reported income*							2<br>0.59	0<br>0.00	0<br>0.00	0<br>0.00	0<br>0.00	2<br>0.33
Third lowest proportion of reported income	69<br>22.77	27<br>16.46	3<br>5.00	1<br>2.08	0<br>0.00	100<br>16.67	81<br>23.82	25<br>18.66	3<br>5.36	1<br>2.50	0<br>0.00	110<br>18.33
	25	11	0	0	0	36	9	3	0	0	0	12

Joint (3 individuals) highest proportion of reported income*	8.25	6.71	0.00	0.00	0.00	6.00	2.65	2.24	0.00	0.00	0.00	2.00
Second highest proportion of reported income	94	20	4	1	0	119	92	23	1	0	0	116
	31.02	12.20	6.67	2.08	0.00	19.83	27.06	17.16	1.79	0.00	0.00	19.33
Joint (2 individuals) highest proportion of reported income*	25	16	1	0	0	42	39	15	0	0	0	54
	8.25	9.76	1.67	0.00	0.00	7.00	11.47	11.19	0.00	0.00	0.00	9.00
Highest proportion of reported income	40	27	7	0	0	74	48	23	3	0	0	74
	13.20	16.46	11.67	0.00	0.00	12.33	14.12	17.16	5.36	0.00	0.00	12.33
**Total**	303	164	60	48	25	600	340	134	56	40	30	600

Notes: In the first and second line of each row, we report, respectively, the per period number and the percentage of whistleblowers' signals on a group member as a function of her relative proportion of reported income within the group. *When there are 2 or more group members reporting exactly the same proportion of their gross income (i.e. 0% or 100%), we are referring to them as the "joint" lowest/highest proportion of reported income.

Appendix B – Experimental instructions and post-experimental questionnaire

The experimental instructions, originally in Italian, were shown on the screen of each participant and were read aloud by the experimenter. Participants were not allowed to proceed with the instructions until the experimenter decided to let them click on the "Next" button. Instructions in black roman refer to the NoWhistle_NoInfo treatment, while specific instructions for the Information treatment are signaled in yellow specific instructions for the Whistleblowing treatments are signaled in *italic*.

Words in square brackets are just meant for the readers of the paper, they were not included in the original instructions.

[New Screen]

Instructions

You are participating in an experiment on decision-making. During this experiment, you can earn money. The amount of your earnings depends on your decisions and the behavior of other participants you will interact with, according to the rules that will be described in what follows.

The whole session lasts about one hour and a half.

Please switch your mobile off and do not talk to each other during the experiment.

What happens now?

We will give you detailed instructions about the experiment. You can raise your hand at any time so that a research assistant will come to your desk and answer your questions in private.

[New Screen]

Introduction

In this experiment, you will be randomly matched with 4 other participants to form a group of 5. You will be matched with the same in-group members for the entire duration of the experiment. You will never know the identity of your group members and all decision will be taken anonymously.

The experiment is composed by two different parts: Part 1 and Part 2. Part 1 consists of 10 periods and Part 2 consists of 5 periods. In each period of Part 1 and of Part 2, you have to make

some decisions. The decisions you take in each period will affect your payoff as well as they might affect the payoffs of other participants who are matched with you. Similarly, the decisions made by participants matched with you, will also affect their payoffs as well as they might affect your payoff.

At the end of the study, we will randomly select the first or the second part with equal probability and, within the selected part, we will randomly select one period. You will be paid in cash the earnings you obtained in that period. Your total earnings from the experiment will be the earnings for the randomly selected period, plus a show up fee of € 3.
You will get paid one by one, in private, in order to preserve confidentiality, on presentation of the ticket that you have randomly drawn from the envelope upon entering the laboratory.
During the experiment we will speak of points rather than Euros.
The conversion rate between points and Euro is: 12 points = 1 euro.

Before giving you detailed instructions about the first part [Information treatments] and the second part of the study, we will resume here the main information about the functioning of [Information treatments] both the first [Information treatments] and the second part part of the experiment:

FIRST PART	SECOND PART
In each period of the first part you and your group members will receive an endowment and you have to privately and anonymously decide how much of it to report. A tax is withdrawn from the reported amount.<br>The reported amount of each in-group member might be subject to an auditing procedure. In case the reported amount is lower than the initial endowment, a fine must be paid.<br>Each individual is informed about other group members' endowment and reported income.<br>Whether each in-group member is audited or not depends on a random procedure.<br>*[Whistleblowing treatment]*	Instructions about the second part will be given once the first part is terminated.<br>[Information treatments]<br>In each period of the second part you and your ingroup members will receive an endowment and have to privately and anonymously decide how much of it to keep with you and how much of it to invest in a project.<br>The amount of points you decide to invest in the project will be doubled and equally divided between the other members of your group, you excluded. Symmetrically, the amount of points that each member of your group decides to invest in the project will be doubled and equally divided

On the base of this information, each individual will have the opportunity to signal the other in-group members. The higher the number of times an individual is signaled, the higher is the probability he will be audited.	between you and the remaining group members.

What happens now?

We will now give you detailed instructions [Information treatments] both about the first [Information treatments] and second part of the experiment. You can raise your hand at any time so that a research assistant will come to your desk and answer your questions in private. Once you have carefully read the instructions, you will be asked to answer a few questions to verify your understanding.

[New Screen]

Instructions - first part

Your choices in the first part.

In each period of the first part, you have to choose which share of income to report in order to pay taxes. In particular, in the first part, the computer will randomly and anonymously assign to each participant an amount of points included between 100 and 240 points, in integer numbers. For simplicity, let us refer to this amount of points as the gross income. Given your gross income, you have to choose how many points to report. Based on the reported income, the computer will collect 30% of it as a tax. The total amount collected will be used by the experimenter for funding future research projects.

Once all your group members have decided their declared income, you are informed about their **real gross** income and the income they have declared.

The amount of points you have chosen to report can be selected for auditing to verify the correspondence of your choice with respect to your gross income.

How payoffs are determined

- In the case your choice is not selected for auditing, then your earnings in the period is given by:

  your gross income **minus** the taxes computed on the amount of points you have reported (30%).

- In the case your choice is selected for auditing and the amount of points you have reported is lower than your gross income, then your earnings in the period is given by:

  your gross income **minus** the taxes computed on your gross income **minus** a fine that is equal to the taxes you have not paid.

Once each participant has reported his own income, each subject in your group is randomly and anonymously assigned one of five cards, numbered from 1 to 5, by the computer. Then, the computer randomly selects one of the 5 cards. The choices made by the owner of this card will be audited. Notice that the probability to be audited in a given period does not depend on the results of the auditing procedures conducted in previous periods.

At the end of each period you are informed about whether you were audited by the central authority and about your payoff for the period.

Whistleblowing

[Whistleblowing treatment]

Once each participant has reported his own income, you and the other group members will choose whether to signal one ingroup members to the central authority in order to be audited.

Once all signaling decisions have been taken, one randomly selected participant is randomly selected and her/his decision is implemented:

- *If, for example, the randomly selected participant decided to signal to the central authority one participant with whom he or she was matched, this participant will be audited by the central authority.*
- *If, for example, the randomly selected participant decided not to signal anyone to the central authority, the central authority's audit decision develops as follows:.*
    - *Each subject in your group is randomly and anonymously assigned one of five cards, numbered from 1 to 5, by the computer. Then, the computer randomly selects one of the 5 cards. The reporting decision made by the owner of this card will be audited. Notice that the probability to be audited in a given period does not depend on the results of the auditing procedures conducted in previous periods.*

At the end of each period you are informed about whether you were audited by the central authority and about your payoff for the period. Please note that your group members are never informed about your signaling decision. Neither you will be informed whether you were selected for auditing randomly or due to information received from some of your group members.

What happens now?

You will be now asked to answer a few questions to verify your understanding about the first part of the experiment.

[Information treatments] Before starting the first period of the first part, we will give you detailed instructions about the second part of the experiment.

[New Screen]

Comprehension questions for the first part

- Suppose a participant received an endowment of 100 points. He/she decided to declare 100 points. He/she was not audited in this period. What will be his/her final earnings for this period?
  100, 70, 40
  [Correct answer: 70]

- Suppose a participant received an endowment of 100 points. He/she decided to report 50 points.

- What's the amount of the fine he/she has to pay in case he/she is audited?
  30, 15, 0
  [Correct answer: 15 (which is equal to the amount of the taxes not paid. When reporting his/her entire gross income, he/she would have paid 30 points in taxes. However, since he/she is reporting 50 points instead of 100 points, he/she just paid 15 points in taxes.)]

- What's the final payoff of the participant if he/she is audited?
  100, 85, 55
  [Correct answer: 55 (which is equal to his/her gross income (100) minus the taxes computed on his/her gross income (30) minus a fine that is equal to the taxes he/she has not paid (15).)]

- What's the final payoff of the participant if he/she is is not audited?
  100, 85, 55
  Correct answer: 85 (which is equal to his/her gross income (100) minus the taxes computed on his/her reported income (15)).

- [NoWhistleblowing treatments] What is the probability for a participant of being audited in each period?
  1/5, 2/5, it depends on my reported income
  [Correct answer: 1/5]

- [NoWhistleblowing treatments] In period 1 a participant was audited and his/her reported income was different than his/her gross income.

- [NoWhistleblowing treatments] What is the probability that this participant will be audited in period 2?
  1/5, 2/5, It depends on his/her reported income
  [Correct answer: 1/5]

- *[Whistleblowing treatments] One group member, let's call him/her subject A, chose to signal to the central authority one of his/her ingroup members, let's call him/her subject B. Subject B decided not to signal anyone to the central authority.*

- *If Subject A is randomly chosen by the central authority as the group member whose signaling decision is implemented, what is the probability that subject B will be audited?*
  Subject B will be audited for sure, 1/5, 0, It depends on his/her reported income
  [Correct answer: Subject B will be audited for sure]

- *If Subject B is randomly chosen by the central authority as the group member whose signaling decision is implemented, what is the probability that subject A will be audited?*
  Subject A will be audited for sure, 1/5, 0, It depends on his/her reported income
  [Correct answer: 1/5]

[New Screen]

What happens now?

The first part of the experiment is about to start. During the experiment, you can raise your hand at any time so that a research assistant will come to your desk and answer your questions in private. If everything is clear please click on "Continue".

[Information treatments] We will now give you detailed instructions about the second part of the experiment. You can raise your hand at any time so that a research assistant will come to your desk and answer your questions in private. Once you have carefully read the instructions, you will be asked to answer a few questions to verify your understanding.

[New Screen]

Second part - Instructions.

Your choices in the second part

In each period of the second part, you and other 4 participants you were matched with in the first part, will receive an endowment of 100 points.

In each period you and other members of your group will have to make a decision how many points to keep with you and how many points invest into a project.

The points you invest in the project is doubled and shared equally between other 4 members of your group, you excluded. Similarly, the amount of points eventually invested in the project by another member of your group, for example by ingroup member A, will be doubled and equally shared between you and the other 3 members of your group, ingroup member A excluded.

How payoffs are determined

In the second part, your earnings in each period are given by:

Your initial endowment **minus the** amount of points you invest in the project **plus** the amount of points your group members invested in the project **multiplied** by 2 and **divided** by **4.**

Example 1:

You decided to invest half of your endowment (50 points) in the project. Other group members in total invested 200. You will receive:

100 - 50 + (200 * 2 / 4) = 150

Example 2:

You decided to invest nothing in the project. Other group members in total invested 200. You will receive:

100 – 0 + (200 * 2 / 4) = 200

What happens now?

You will be now asked to answer a few questions to verify your understanding about the second part of the experiment.

Once you have correctly answered to these questions, the second part of the experiment will start. During the experiment, you can raise your hand at any time so that a research assistant will come to your desk and answer your questions in private. If everything is clear please click on “Continue”.

[Information treatments] Once you have correctly answered to these questions, the first part of the experiment will start. During the experiment, you can raise your hand at any time so that a

research assistant will come to your desk and answer your questions in private. If everything is clear please click on “Continue”.

[New Screen]

Comprehension questions for the second part

- Suppose subject A invested his/her entire endowment in the project. The other members of his/her group invested nothing.
- What will be group member A's final earnings for this period?
  0, 100, 150, 200, 300
  [Correct answer: 0]
- What will be A's in group members' final earnings for this period?
  0, 100, 150, 200, 300
  [Correct answer: 150]
- Suppose subject A invested nothing in the project. Each of the other 4 members of his/her group invested their entire endowment (100 points). What will be subject A's final earnings for this period?
  0, 50, 100, 200, 300
  [Correct answer: 300]
